# Abdominal erythema ab igne—An "old" finding revisited

**DOI:** 10.1002/ccr3.2834

**Published:** 2020-04-16

**Authors:** Igor Alexander Harsch

**Affiliations:** ^1^ Department of Internal Medicine II Division of Endocrinology and Metabolism Thuringia Clinic Saalfeld "Georgius Agricola" Saalfeld Germany

**Keywords:** erythema ab igne, Merkel cell carcinoma, pancreatitis, squamous cell carcinoma, toasted skin syndrome

## Abstract

Abdominal erythema ab igne may be the result of insufficient pain medication in chronic pancreatitis. Stenting therapy is to be considered, and the patient should be informed about the risk of developing cutaneous squamous or Merkel cell carcinoma.

## Graphical abstract

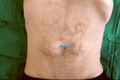

## 1 CASE REPORT

Abdominal erythema ab igne has become a rare finding owing to the progress in the therapy of chronic pancreatitis. Due to the risk of developing dermal squamous cell or Merkel cell carcinomas, the finding has to be taken seriously.

**Figure 1:**
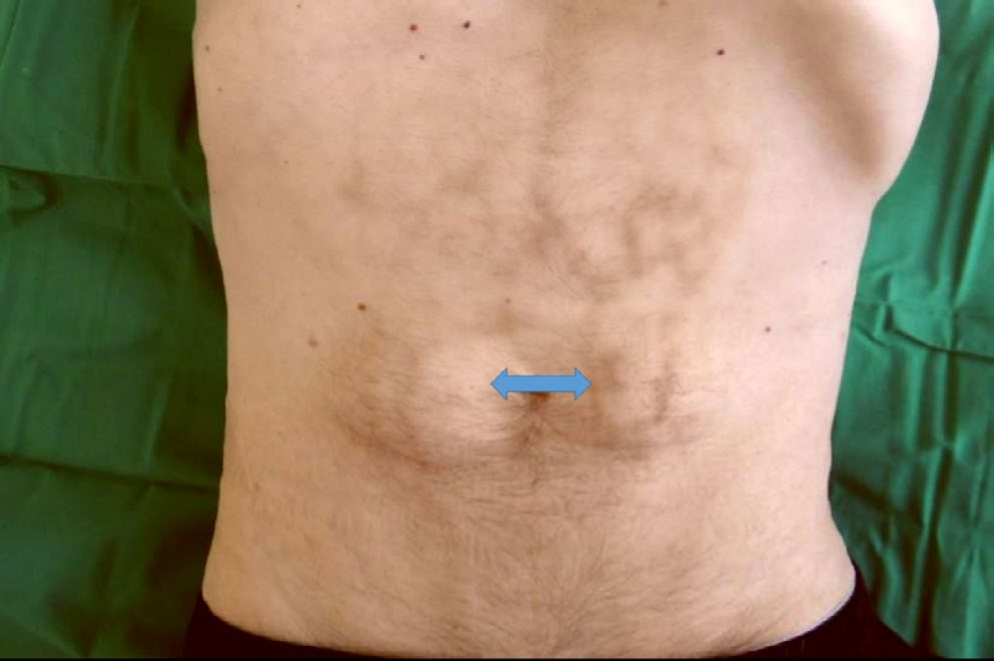
View of the patient's abdomen: Net‐like skin drawing (erythema ab igne) and 2 symmetrical protrusions of fatty tissue (lipohypertrophies due to repeated insulin injections in these regions, see blue arrows)

After years of nicotine and alcohol abuse, a 58‐year‐old patient had chronic pancreatitis with endocrine and exocrine insufficiency. He was introduced to an endoscopic stenting therapy. Physical examination showed a net‐like skin drawing of the abdomen (Figure 1). The patient treated painful episodes by using a hot‐water bottle, and pain relief due to tramadol and amitriptyline was not always sufficient. He does not feel the heat (diabetic and ethylic neuropathy?).

These cutaneous findings are called "erythema ab igne" (Latin: ignis = fire). The repeated heat damage of the skin leads to atrophy, persistent reticular hyperpigmentation, and telangiectasia^1^ and is a risk factor for developing squamous cell or Merkel cell skin carcinomas.

**Figure 2:**
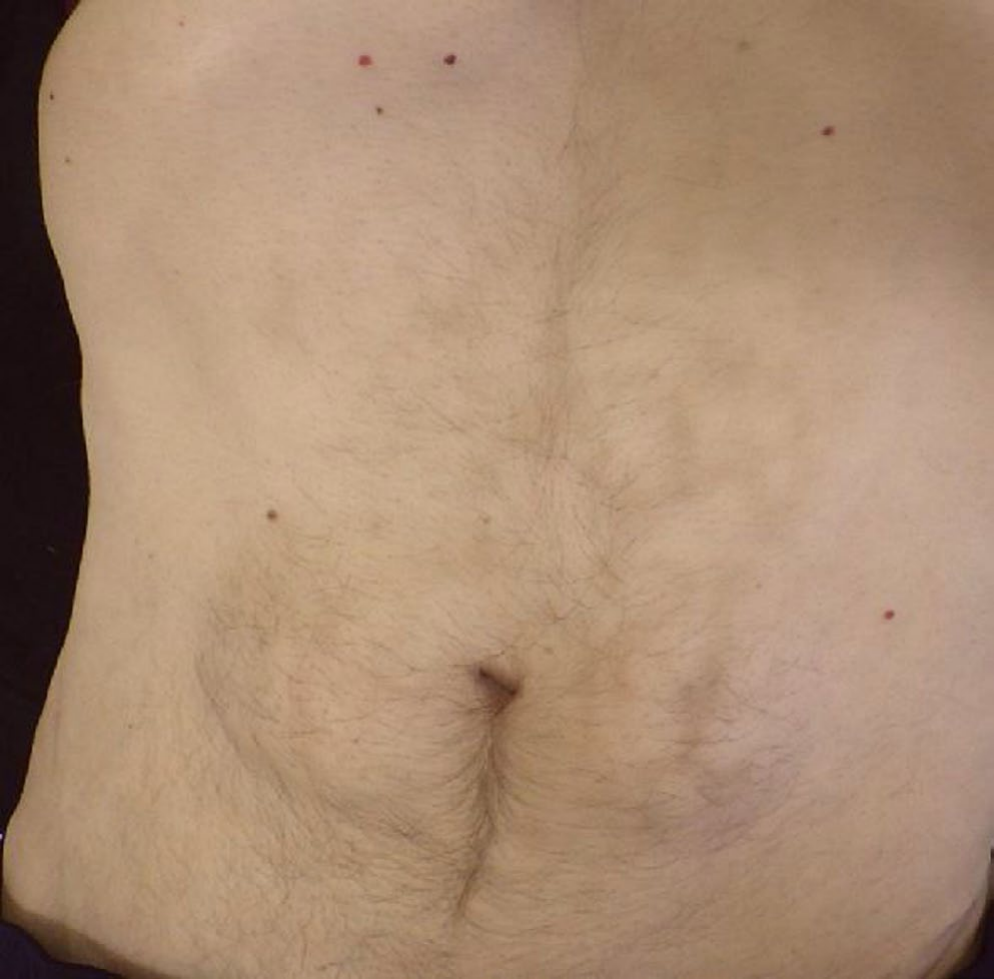
View of the patient's abdomen, three months later: The net‐like skin drawing has faded significantly

The finding became rare in recent decades due to advances in the endoscopic treatment of chronic pancreatitis and pain therapy. Stenting therapy was successful in relieving pain over time and the patient renounced from self‐treatment. Three months after the first stenting, hardly any cutaneous lesions were detectable (Figure 2). Nowadays, abdominal erythema ab igne has become rare, but such lesions are reported after work with the laptop and placing it on the thighs for extended periods of time on numerous occasions.^2^

## ETHICS STATEMENT

The patient gave written consent to report his case and the imaging.

## CONFLICT OF INTEREST

None declared.

## AUTHOR CONTRIBUTIONS

IAH wrote the article and has accountability for all aspects of the work.
